# Olive Garden’s Expansion Of Paid Sick Leave During COVID-19 Reduced The Share Of Employees Working While Sick

**DOI:** 10.1377/hlthaff.2020.02320

**Published:** 2021-08

**Authors:** By Daniel Schneider, Kristen Harknett, Elmer Vivas-Portillo

**Affiliations:** Harvard Kennedy School, Harvard University, in Cambridge, Massachusetts.; Department of Social and Behavioral Sciences, University of California San Francisco, in San Francisco, California.; analyst at the Center for Effective Philanthropy, in San Francisco, California. He was a fellow at the Harvard Kennedy School when this work was performed.

## Abstract

The COVID-19 pandemic has focused public and policy attention on the acute lack of paid sick leave for service-sector workers in the United States. The lack of paid sick leave is potentially a threat not only to workers’ well-being but also to public health. However, the literature on the effects of paid sick leave in the US is surprisingly limited, in large part because instances of paid sick leave expansion are relatively uncommon. We exploit the fact that large firms in the US were not required to expand paid sick leave during the COVID-19 pandemic but that one casual dining restaurant in particular, Olive Garden, faced intense public pressure to do so. We drew on data collected from 2017 through fall 2020 from 10,306 food service–sector workers in the US by the Shift Project, which include employer identifiers. Using a difference-in-differences design, we found strong evidence of an increase in paid sick leave coverage among Olive Garden workers, as well as evidence that this expansion reduced the incidence of working while sick among front-line food service workers.

The United States is one of two countries in the Organization for Economic Cooperation and Development that does not guarantee paid sick leave to employees. In contrast, every European Union Member State offers employees the right to paid sick leave and sick pay.^1^ Without a federal policy, access to paid sick leave in the US varies by city and state or is at the discretion of employers, resulting in stratification by work sector and socioeconomic status.^2–4^

The COVID-19 crisis has brought this lack of paid sick leave to the forefront of public and policy attention, with a particular focus on service-sector workers. These include the front-line and “essential” workers who have continued to stock stores, fulfill takeout orders, and deliver necessities throughout the health crisis. Yet according to the 2019 National Compensation Survey from the Bureau of Labor Statistics, only 58 percent of service workers reported access to paid sick leave that year, compared with more than 90 percent of workers in management, business, and finance.^5^ Across the wage distribution, fewer than 50 percent of low-income and part-time workers had access to paid sick leave, compared with 90 percent of workers with the highest wages.^5^

In response to COVID-19, the US federal government adopted a temporary national paid sick leave policy, the Families First Coronavirus Response Act. This law, which was effective from April 1, 2020, through December 31, 2020, required certain employers to provide paid sick leave to employees who may have contracted the virus or who needed to self-isolate or quarantine, to care for a family member with COVID-19, or to care for a child whose school had closed.^6^ This law effectively reduced COVID-19 transmission.^7^ However, the law exempted employers with more than 500 or fewer than 50 employees.

These gaps in paid sick leave coverage have real consequences for worker and public health.^8,9^ Quasi-experimental research shows that in cities and states that have expanded paid sick leave mandates, coverage rates increase.^10,11^ The expansion of paid sick leave has led to mixed findings on the association between these laws and leave taking: Although some researchers find reductions in leave taking, presumably because reduced disease transmission lessens the need for leave,^12^ others find increases, attributing these potentially to newly covered workers now being able to take time off.^10,11^ For populations without previous access to paid sick leave, who are disproportionately low wage and in the service sector, there is some evidence that presenteeism significantly declines after paid sick leave expansion.^11,13^ One study found that workers without paid sick leave are more likely to report to work when contagious.^14^ However, once employees have access to paid sick leave, community rates of influenza-like illness significantly decrease.^7,15^

Although the Families First Coronavirus Response Act did not mandate expansion of paid sick leave by large firms, such firms have found themselves the subject of media attention focused on the insufficiency of their paid sick leave coverage.^16,17^ Perhaps most prominently, Olive Garden (owned and operated by Darden Restaurants Inc.) was the subject of a piece of investigative journalism focused on its lack of paid sick leave coverage for workers.

On March 9, 2020, journalist and activist Judd Legum published an exposé of the lack of paid sick leave at Olive Garden, a large, multistate, casual dining company that is among the largest food-service companies in the US.^18^ Legum’s story was widely shared on social media, and on the same day it was published, Darden announced that it would substantially expand its paid sick leave, pledging to credit employees with one hour of paid sick leave for every thirty hours worked and applying this accrual formula retroactively over the prior twenty-six weeks.^19^

However, just because a firm announces a change to corporate policy does not mean that the policy change will actually be implemented. For instance, in 2014, after its precarious scheduling practices were featured in a widely read *New York Times* article,^20^ Starbucks pledged change^21^ but was then exposed as having done little to resolve the issue.^22^ Similarly, in 2016 Walmart announced that it would provide more stable and predictable schedules to its workers,^23^ but follow-up work three years later showed that it had made little progress toward making that change a reality.^24^

This apparent mismatch between what firms say they will do in response to negative public attention and what firms actually do for workers casts some doubt on the efficacy of investigative journalism for precipitating real change for workers. Indeed, advocates and social movement scholars are often skeptical that online-only campaigns against firms can be effective without a significant investment in worker organizing and leadership development.^25–27^ However, it has been difficult to examine empirically whether such press attention and online campaigns do in fact change employers’ policies on the ground because few matched employer-employee data exist in the United States that would permit such analysis.

In this article we make two key contributions to the literature on paid sick leave. First, we estimated the effects of the media coverage of Olive Garden’s lack of paid sick leave policy and of the company’s announcement on the actual experiences of workers. Second, as a unique complement to existing work on paid sick leave that uses legislated expansions in coverage,^4,7,11,13,15^ we studied whether this nonlegis-lated change in corporate practices reduced presenteeism among workers. One key advantage of this approach is that we focused on workers who have very limited access to paid sick leave, but who, as front-line food service workers, likely play a key role in disease transmission.^11,28^

To do so, we drew on new employer-employee matched data from the Shift Project, collected from 2017 through November 2020 from 10,306 hourly workers employed at Olive Garden. We estimated difference-in-differences models to reveal the effects of this campaign on employees’ access to paid sick leave and on employees working while sick, which we refer to as worker presenteeism.We found that the campaign against Olive Garden was highly effective, increasing employee-reported access to paid sick leave by 49 percentage points. We further found that employers’ expansions of paid sick leave during March–May 2020 significantly reduced presenteeism by September–November.

## Study Data And Methods

### Data Source

This article draws on data from the Shift Project, an ongoing survey of service-sector workers at large retail and food service firms across the US. The Shift Project uses Facebook as both a sampling frame and a survey recruitment device. The pool of potential respondents is identified and then recruited from large, named retail and food service companies, using targeted advertisements on Facebook and Instagram. Survey recruitment advertisements target adults who are ages eighteen and older, living in the US, and employed at one of 142 identifiable large retail or food service firms. This set of 142 firms represents the largest retail and food service establishments by revenue and employs more than 50 percent of the entire service-sector workforce.^29^

The Shift Project fields repeated cross-sectional surveys of workers employed at these firms. Beginning with the third wave of data collection (fielded September–November 2017), the survey asked respondents whether they could receive paid sick days at their job. This question was included on six subsequent waves (wave 4, February–June 2018; wave 5, October–November 2018; wave 6, February–April 2019; wave 7, September–November 2019; wave 8, March–May 2020; and wave 9, September–November 2020). In total, there are data on 66,703 respondents across these seven waves. Workers at a subset of thirty large firms were sampled at each of the seven waves.Workers at an additional 112 firms were sampled intermittently. Workers at Olive Garden were surveyed at waves 3, 5, 6, 8, and 9. Therefore, we restricted our sample to 50,654 workers surveyed at these 112 firms across those five waves. We further limited our analytical sample to the 16,366 workers who were employed during these waves at the 46 food service firms included in the data set. We then eliminated cases with missing data on our outcomes or covariates, which produced a final analytic sample of 10,306 workers employed at food service firms, including 653 Olive Garden workers. In supplementary analyses we limited the sample to 2,562 workers at the fifteen casual dining employers included in the data set. The sample size of workers at each employer is listed in supplemental exhibit 1 in the online appendix, with the casual dining employers in boldface.^30^

We constructed survey weights to adjust the demographic characteristics of the Shift Project survey sample to match the demographic characteristics of service-sector workers in the Census Bureau’s American Community Survey. After we applied these weights, the demographic characteristics of our survey sample closely aligned with the respective benchmark sample.^31^

### Measures

- **PAID SICK LEAVE:** We analyzed paid sick benefits across waves, using the following survey question: “Please look at the following list of benefits that employers sometimes make available to their employees.Which of the benefits on this list can you receive as part of your job at [employer name]? Please mark all that apply.” Among the ten listed options was “Paid sick days.” Respondents who indicated that they could receive paid sick days were coded as 1, whereas those who did not were coded as 0.
- **PRESENTEEISM:** To measure presenteeism, we made use of the question, “In the past month, did you ever work at [name of employer] even though you were feeling sick?” Respondents could select “Yes,” “No, I was sick but I stayed home,” or “No, I haven’t been sick in the past month.” This variable was then coded as 0 if the respondent either reported not feeling sick or reported feeling sick but staying home. The variable was coded as 1 if the respondent reported going to work despite feeling sick.
- **CONTROL VARIABLES:** We controlled for a set of demographic characteristics that could bias the association between paid sick leave and presenteeism.We adjusted for the respondent’s sex, race/ethnicity, and age; whether the respondent had children; whether the respondent spoke a language other than English at home; whether the respondent was currently enrolled in school; the respondent’s educational attainment; and whether the respondent was married or living with a partner.

We also controlled for a set of work characteristics including whether the respondent was a manager, how many years the respondent had been at their job, the respondent’s hourly wage, what shift the respondent worked (regular day shift, regular night shift, regular evening shift, a variable shift, or a rotating or split shift), if the respondent was working involuntarily part time, and who controlled the respondent’s schedule (employer decides starting and stopping times, employer decides with respondent input, or respondent mostly decides).

### Analytic Methods

We estimated a difference-in-differences model that compared workers at Olive Garden before and after the media coverage with workers at other large food service employers.We compared Olive Garden workers with the set of workers at all other fast food, coffee shop, and casual dining employers represented in the Shift Project data. We also tested the robustness of our result by restricting the comparison sample to only those workers at other casual dining chains.

We estimated the difference-in-differences model using a linear probability model, with the compositional controls described above, weighted to the American Community Survey, and with robust standard errors that accounted for employer clustering. We then estimated the same difference-in-differences model with presenteeism as the outcome.

Olive Garden’s introduction of paid sick leave allowed for longer-tenured workers to receive paid sick leave credit for hours worked during the prior year. If this practice worked as announced, then we would expect that any reduction in presenteeism would be larger for longer-tenured workers. Therefore, we reestimated the difference-in-differences model for workers with at least two years of tenure.

Our analysis tested whether an expansion of paid sick leave led to reductions in presenteeism. A competing possibility is that wage increases might have allowed for workers to more easily afford unpaid time off. We exploited the rich set of measures in the Shift Project data to test whether any other aspects of job quality showed similar divergence from trend after the public campaign. To do so, we estimated the same difference-in-differences model, taking wages, usual work hours, involuntary part-time work, exposure to unstable scheduling, paid vacation, medical insurance, and retirement plan as outcome variables. We found little evidence of any other changes in job quality and nothing on the scale of the change in paid sick leave access. We present these results in supplemental exhibit 2.^30^

### Limitations

The study had several limitations related to sampling and selection. The Shift Project data are collected using a nonprobability sampling approach. One potential threat to inference is if there is selection into the use of Facebook and Instagram. Here, data from Pew Research using traditional polling suggest that this is unlikely to be a major source of bias. Large shares of employed Americans are regularly active on these platforms, and there is not significant variation by educational attainment or household income.^32^

Another potential source of bias is selection into the survey conditional on being a platform user. Approximately 1.2 percent of users who were presented with the recruitment advertisements contribute survey data. Respondents may select unequally into the survey on the basis of characteristics such as sex, race/ethnicity, age, or education. Supplemental exhibit 3 compares the demographics of the Shift Project sample with those of workers in the same set of industries and occupations in the American Community Survey and shows that women and White, non-Hispanic respondents are overrepresented in our sample.^30^

Our survey weights corrected for this demographic overrepresentation, but it is also possible that respondents select into the survey on the basis of unobservable characteristics that could be confounders. Although such selection cannot be ruled out, the Shift Project data closely replicate associations in other “gold standard” data sets, such as the wage returns to job tenure in the Current Population Survey and National Longitudinal Survey of Youth.^31^ Further, for the analysis undertaken here, the analytical design went some way toward mitigating these concerns. As described above, by comparing Olive Garden workers in the Shift Project data set with other food service workers in the same data set and comparing those two groups over time, we narrowed the scope of problematic selection. Respondents would need to be differentially selected into the survey by firm and by time to bias the results.

## Study Results

Exhibit 1 plots estimates from the difference-in-differences model of the share of workers who reported paid sick leave at Olive Garden and at all other food service employers. In the years before COVID-19 (2017, 2018, and 2019), we see that very few workers at Olive Garden, about 23 percent, reported access to paid sick leave and that this share was in fact slightly higher, at 30–35 percent, at comparison employers. Access to paid sick leave stayed about the same from 2017 through 2019 both at Olive Garden and at other food service employers.

However, after Olive Garden’s announcement, this situation changed dramatically. Although workers at comparison firms saw no real change in their access to paid sick leave, the share of Olive Garden workers reporting access increased substantially and significantly from 23 percent in spring 2019 to 66 percent in spring 2020. This expansion was persistent into fall 2020, with 64 percent of Olive Garden workers reporting access to paid sick leave, compared with 29 percent of comparison workers. After we parceled out the secular trend in paid sick leave estimated for the comparison workers, the difference-indifferences estimates were 49-percentage-point increases in paid sick leave for Olive Garden workers (*β* = 0:49; *p* < 0:001) in spring and fall 2020. These difference-in-difference estimates are the coefficients from the interactions between survey wave and employer, presented in exhibit 2, model 1.

As a robustness check, supplemental exhibit 4 shows the results of conducting the same analysis but comparing Olive Garden only with other casual dining employers, with the same pattern of results.^30^ In supplemental exhibit 5, we present coefficients from the models that demonstrate the robustness of these estimates to alternative model specifications.^30^

Exhibit 2, model 2 presents estimates from the difference-in-differences model of presenteeism, comparing Olive Garden workers with all other food service workers. As access to paid sick leave increased significantly in spring 2020, we found that the reduction in presenteeism did not manifest until fall 2020. Calculating predicted values from these estimates, in fall 2020, 28 percent of comparison food workers reporting working while sick, compared with 16 percent of Olive Garden workers. After parceling out the secular trend in presenteeism for comparison workers, we estimated a significant 15-percentage-point reduction in the share of Olive Garden workers who reported working while sick in the prior month.

Exhibit 3 plots the predicted values of presenteeism from model 2 of exhibit 2. In the pre-COVID-19 periods (fall 2017–fall 2019), we estimated very high levels of self-reported presenteeism, with approximately 55 percent of workers at Olive Garden reporting that they were sick but went to work anyway at least once in the prior month. These levels far exceed prior estimates for the percentage of food service workers reporting to work while ill.^33–35^ One explanation is that prior research has specifically focused on workers going in when experiencing symptoms associated with foodborne illnesses, such as vomiting, diarrhea, or a stomach flu, whereas the Shift Project survey asks about working while sick more broadly. Another possibility is that respondents to the Shift Project survey, although asked about the prior month, are in fact reporting on a longer recall period (“forward telescoping”), a common occurrence when reference periods are of short duration.^36^

However, for the purposes of identifying the effects of paid sick leave expansion on presentee ism, the levels are less important than the similarity of the pre trends for Olive Garden workers and comparison food service workers. Here, we found nearly identical trends in presenteeism in the pre-COVID-19 period. Notably, in the early months of COVID-19, March–June 2020 (spring 2020 in exhibit 3), we found a marked decline in presenteeism for both Olive Garden workers and comparison workers. This is consistent with a normative shift in the acceptability of presenteeism in the midst of a pandemic.^37,38^ We then observed significant divergence in presenteeism between Olive Garden workers, whose rate continued to decline, and comparison workers in fall 2020.

To test the robustness of this result, supplemental exhibit 6 presents the same analysis but compares Olive Garden workers only with other casual dining workers.^30^ Supplemental exhibit 7 presents coefficients that show the robustness of these estimates to alternative model specifications.^30^

In exhibit 2, model 3 we limited the sample to workers with at least two years of job tenure, who would have had time to accrue paid sick leave. For these workers, we observed large and significant effects of paid sick leave on presenteeism, and those benefits appeared earlier than in the model 1 results, with significant reductions in presenteeism for these workers in spring 2020 that continued into fall 2020.

## Discussion

The COVID-19 pandemic has brought renewed attention to the acute lack of paid sick leave faced by millions of service-sector workers. Although a patchwork of states and localities have passed laws to require paid sick leave coverage, there is no federal requirement that employers do so. Even as Congress passed legislation requiring medium-size businesses to offer paid sick leave during COVID-19 (a law that has since lapsed), employees at small and large firms remained uncovered.Yet even firms not required to expand paid sick leave by law have come under significant public pressure to do so voluntarily. Do such campaigns and press coverage actually affect job quality for workers?

We investigated whether a public campaign against Olive Garden’s lack of paid sick leave led to expanded access to paid sick leave among company employees. We showed that this campaign resulted in a large increase in paid sick leave coverage for Olive Garden workers and that this increased access was persistent across at least nine months. This voluntary increase in paid sick leave coverage was large, even compared with the increases precipitated by state and local mandates,^4,10,11,13^ likely because coverage rates were so low before the change.

Although most prior work on the effects of paid sick leave on presenteeism and public health has used legislatively mandated expansions of paid sick leave, we provide novel estimates of the effect of paid sick leave on presenteeism, using a voluntary company expansion. We found that Olive Garden’s expanded paid sick leave reduced presenteeism by approximately 15 percentage points, with the largest effects being for workers who had two or more years of tenure with the company.We expect that the 49-percentage-point expansion in paid sick leave access played a large role in driving this reduction in presenteeism, but we note that the media attention to this issue may also have induced behavioral changes in Olive Garden workers, which reduced their likelihood of working while sick.

Our estimate of the reduction in presenteeism after an expansion of paid sick leave is broadly consistent with recent research on changes in paid sick leave laws. Using local paid sick leave mandates, Kevin Callison and Michael Pesko estimate that a 7-percentage-point increase in paid sick leave access led to a 4.6-percentage-point increase in work loss days resulting from illness for the workers most affected by legislated expansions of paid sick leave.^11^ Daniel Schneider’s estimates that Washington State’s law increased paid sick leave access by 28 percentage points for service-sector workers and reduced presenteeism by 8 percentage points^13^ are proportionately quite close in magnitude to our estimates of a nearly 50-percentage-point increase in access to paid sick leave and a 15-percentage-point reduction in presenteeism.

Our novel approach to identification, leveraging changes to company policies rather than changes to paid sick leave laws, has considerable promise given the sheer number of firms operating in the service sector and the scope of control that US employers have over job quality. Indeed, in recognition of this power, advocates not only press for legislation to improve job quality but often wage campaigns urging employers to change their practices.^39–41^ We were able to verify whether changes to company practice actually materialize after companies announce changes to policy by using data from the Shift Project from large samples of workers nested within identifiable firms. We have shown that at least in this case, online investigatory journalism coupled with social media activity led to substantial changes in corporate practices, with significant follow-on benefits to workers.

Our work was subject to some important limitations, which also point the way to future research. First, although our data are unique in capturing access to paid sick leave and presenteeism among Olive Garden workers and their counterparts, we lacked a measure of workers actually taking paid sick leave, which would have been a valuable complementary measure. Second, we examined very short-run effects of paid sick leave expansions on presenteeism. It would be valuable to trace the longer-run consequences for presenteeism to see whether gains were sustained or whether they grew or receded over the longer term. Third, we estimated the effects of paid sick leave expansion during the COVID-19 pandemic. On the one hand, we might suspect that the sheer accumulation of challenges and hardships faced by service-sector workers might dampen the effects of an expansion of paid leave. On the other hand, we might also expect paid sick leave expansions to be particularly salient and impactful during COVID-19. Expansions of paid sick leave during nonpandemic periods could play out differently in their effects on presenteeism.

We provide new evidence on the effects of paid sick leave expansion on worker outcomes. It reduced presenteeism, which promotes worker and public health. But only some workers were able to reap these benefits, as federal mandates for paid sick leave coverage exempted employers with more than 500 or fewer than 50 employees, leaving millions of essential workers uncovered. As policy makers consider ways to close these gaps in coverage, one concern is that universal paid sick leave could exert downward pressure on employment or wages. However, prior studies generally find no significant changes to employment or wages,^42^ or if there are effects, they are only modest.^43,44^ On balance, our research adds to the growing body of work showing the benefits of paid sick leave for public health and workers’ well-being—research that builds the evidence base for extending paid sick leave coverage to all workers in the US.

## Supplementary Material

Supplement

## Figures and Tables

**Exhibit 1:**
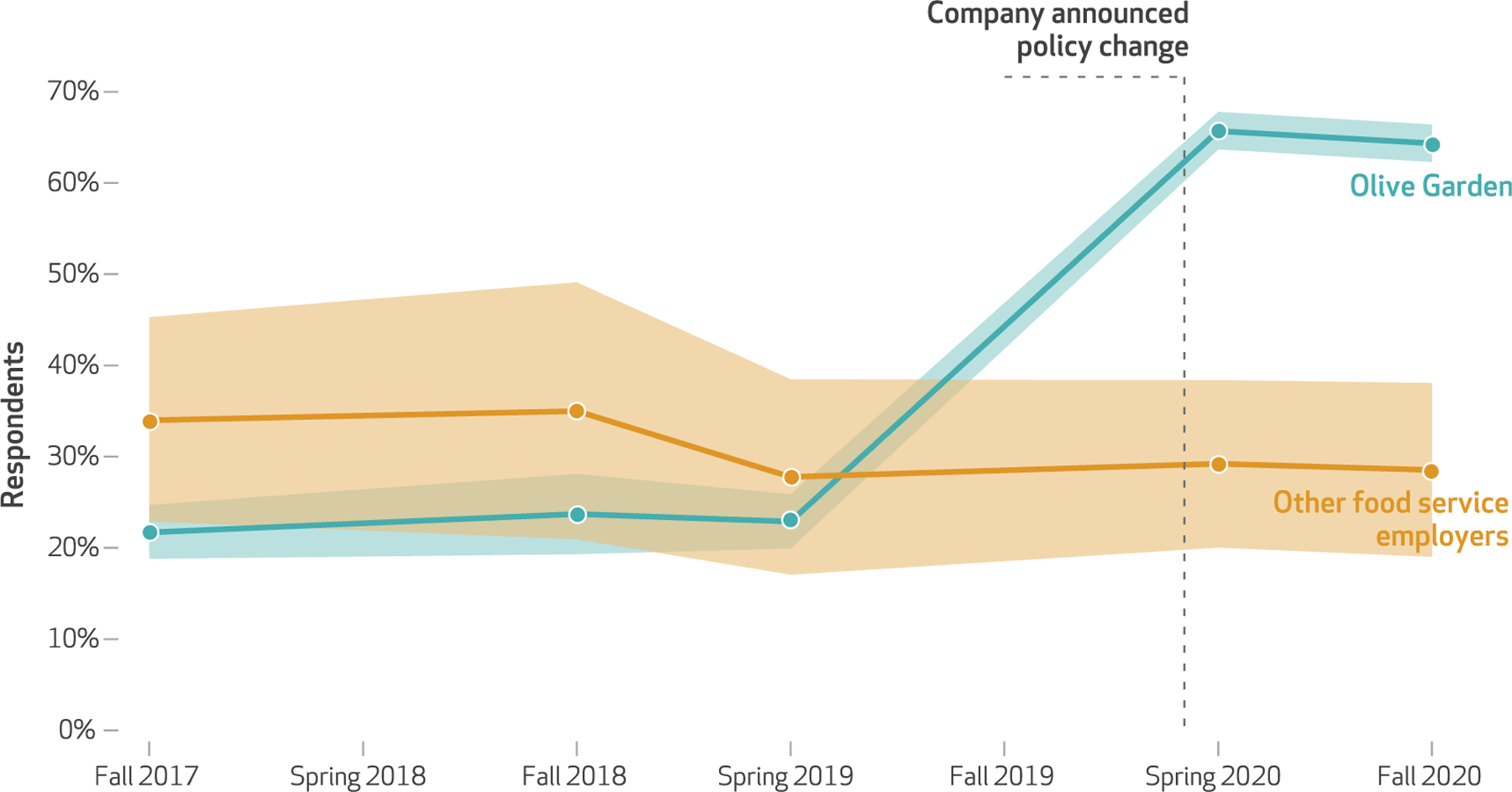
Difference-in-differences estimates of paid sick leave access at Olive Garden versus other food service employers, 2017–20 **SOURCE** The Shift Project data. **NOTES** Model is estimated with survey weights and includes controls for the respondent’s sex, race/ethnicity, and age; whether the respondent had children; whether the respondent spoke a language other than English at home; whether the respondent was currently enrolled in school; the respondent’s educational attainment; whether the respondent was married or living with a partner; whether the respondent was a manager; how many years the respondent had been at their job; the respondent’s hourly wage; what shift the respondent worked; who controlled the respondent’s schedule; and the respondent’s involuntary part-time status. Estimates are connected, but no data are observed between estimated points. The months constituting each data collection point, representing survey waves 3–9, are described in the text.

**Exhibit 3:**
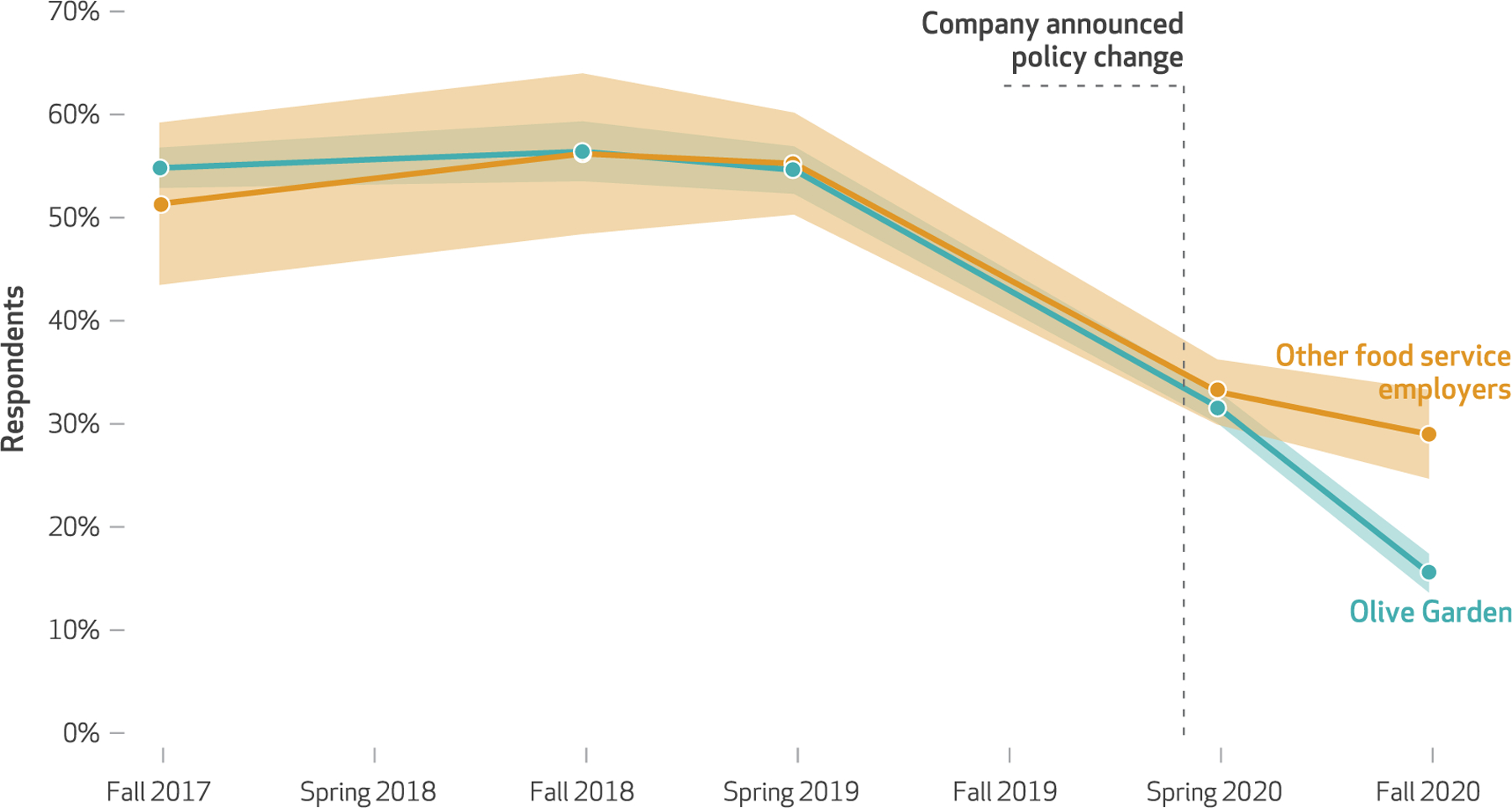
Difference-in-differences estimates of presenteeism at Olive Garden versus other food service employers, 2017–20 **SOURCE** The Shift Project data. **NOTES** Model is estimated with survey weights and includes controls as defined in the exhibit 1 notes. Estimates are connected, but no data are observed between estimated points. The months constituting each data collection point, representing survey waves 3–9, are described in the text.

**Exhibit 2:** Difference-in-differences estimates of paid sick leave access and presenteeism at Olive Garden versus other food service employers, 2017–20

	Access to paid sick leave	Presenteeism	
Interaction term	Model 1: all workers (*N* = 10,306)	Model 2: all workers (*N* = 10,306)	Model 3: at least 2 years of job tenure (*n* = 6,377)
Olive Garden × fall 2017	Ref	Ref	Ref
Olive Garden × fall 2018	0.012	−0.034	−0.064
Olive Garden × spring 2019	0.077	−0.041	−0.062
Olive Garden × spring 2020	0.488****	−0.050	−0.104***
Olive Garden × fall 2020	0.485****	−0.150****	−0.212****

**SOURCE** The Shift Project data.

**NOTES** The variables listed (interaction terms) are the interactions between survey wave and employer; the coefficients are the difference-in-differences estimates as described in the text. All models are estimated with survey weights and include controls as defined in the exhibit 1 notes. The months constituting each data collection point, representing survey waves 3, 5, 6, 8, and 9, are described in the text.

*** *p* < 0:01

**** *p* < 0:001
