# Supplementary material for: Olive Garden’s Expansion Of Paid Sick Leave During COVID-19 Reduced The Share Of Employees Working While Sick
Source: Health Aff (Millwood). Author manuscript; Available in PMC 2022 May 19. (PMC9118176; doi:10.1377/hlthaff.2020.02320)
Supplement: Supplement [file NIHMS1752194-supplement-Supplement.pdf]

APPENDIX

Supplemental Exhibit 1: Restaurants & Other Food Services Firms Sample Sizes

Firm	Count	Firm	Count
**Applebees**	**281**	KFC	144
Arby's	284	Little Caesars	43
**Bob Evans**	34	McDonald's	757
**Buffalo Wild Wings**	**475**	**Olive Garden**	**653**
Burger King	47	**Outback Steakhouse**	**13**
Carl's Jr.	473	**P.F. Chang's**	**156**
Checker's Drive-In	104	Panda Express	421
**Cheesecake Factory**	**566**	**Panera**	**242**
Chick-Fil-A	357	Papa John's	485
**Chili's**	16	Pizza Hut	1
Chipotle	121	Rally's	95
**Cracker Barrel**	**63**	**Red Lobster**	**58**
Culvers	529	Red Robin	178
Dairy Queen	1	Sonic	839
**Denny's**	**483**	Starbucks	7
Domino's	53	Subway	618
Dunkin Donuts	42	Taco Bell	358
Five Guys	84	**Texas Roadhouse**	**5**
Hardee's	198	**Waffle House**	**201**
**IHOP**	**149**	Wendy's	397
In-N-Out Burgers	1	Whataburrger	36
Jack in the Box	4	Zaxby's	77
Jason's Deli	154		
Jimmy John's	281		
N			**10,306**

Note: Casual dining chains are in bold

Supplemental Exhibit 2: Difference-in-Difference Estimates of Job Quality at Olive Garden versus Other Food Services

We consider whether other changes to job quality that were unique to Olive Garden could have contributed to the reduction in presenteeism. In Supplemental Exhibit 7, we show results from a set of models that test whether the campaign might have also led to other improvements in job quality at Olive Garden. The top left panel (red border) reproduces the estimates of the campaign on PSL coverage at Olive Garden. We see some small divergence in wages at Olive Garden, which began to rise in Spring of 2019 and brought wages on par with other employers by Fall 2020. For other indicators of job quality, Olive Garden workers followed a trend similar to comparison workers or experienced slight declines in job quality relative to their counterparts. a slight reduction in usual work hours at Olive Garden in the Fall of 2020, and an increase in retirement plan access in the Fall of 2020. Other aspects of job quality, including schedule instability, paid vacation time, medical insurance, and involuntary part-time work did not change over the period. Across these measures of job quality, PSL clearly stands out for the large and sustained divergent increase.

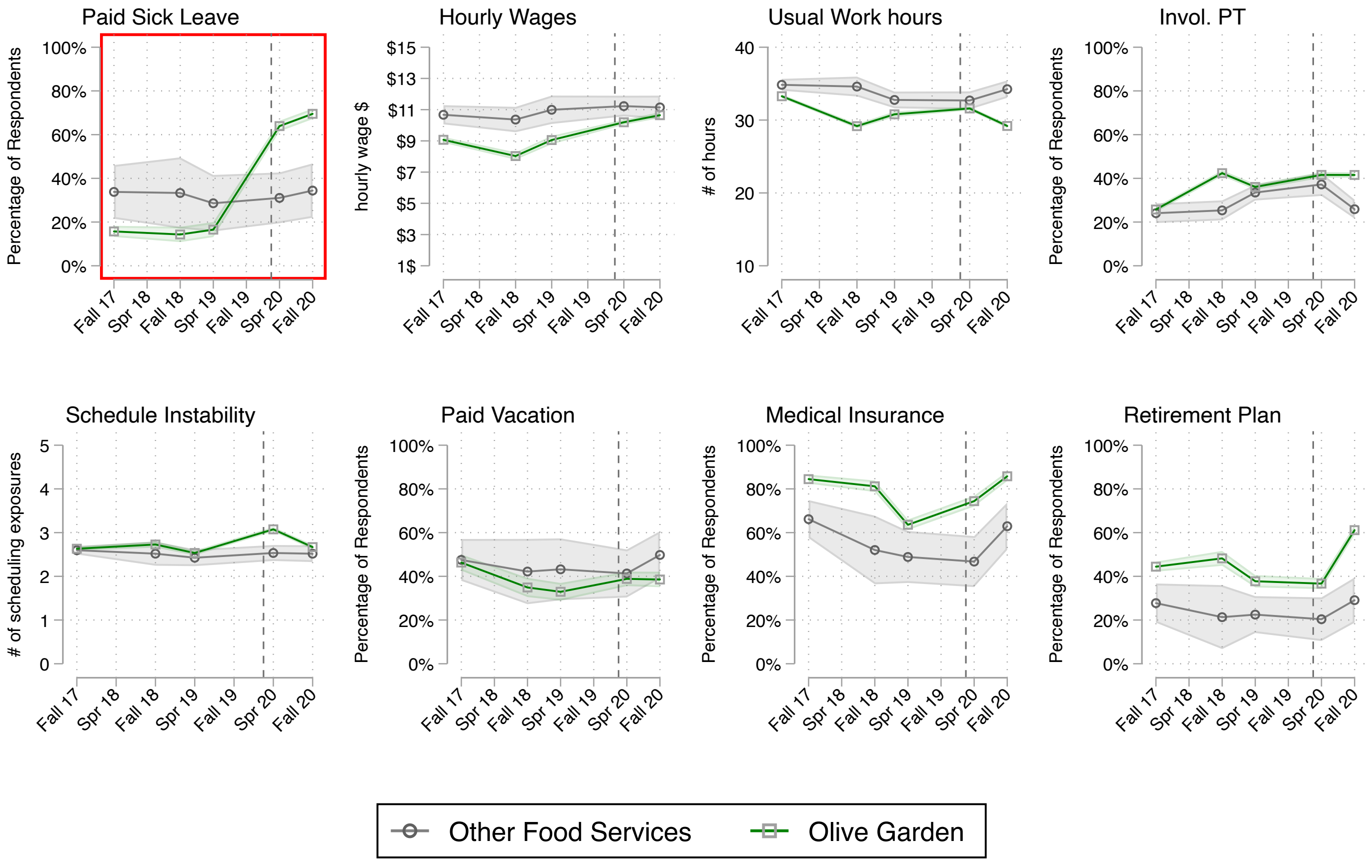

Note. All models are estimated with survey weights and include controls for gender, race/ethnicity, age, whether the respondent has children, whether the respondent speaks a language other than English at home, whether the respondent is currently enrolled in school, respondent's educational attainment, whether the respondent is married or living with a partner, whether respondent is a manager, how many years a respondent has been at their job, and who controls the respondent's schedule. Vertical dashed gray line designates estimated date of PSL policy change. Estimates are connected, but no data are observed between estimated points.

Supplemental Exhibit 3: Demographic Comparison of Shift Project Sample and ACS Respondents in Food and Retail Industries

	ACS	Shift	
		Unweighted	Weighted
Gender			
Male	50%	71%	26%
Female	50%	29%	74%
Race/Ethnicity			
White, non-Hispanic	55%	78%	55%
Black, non-Hispanic	10%	4%	10%
Hispanic	26%	11%	26%
Other/Two or more races, non-Hispanic	9%	7%	9%
Age			
Mean	31	31	31
Median	27	26	26
N	556,403	10,306	10,306

Note: American Community Survey (ACS) data pooled from 2008-2018 and restricted to workers in industry code 641 (eating and drinking places) and occupation codes 434-444 (food Preparation and Service Occupations).

Supplemental Exhibit 4: Difference-in-Difference Estimates of Paid Sick Leave Access at Olive Garden versus Other Casual Dining Restaurants

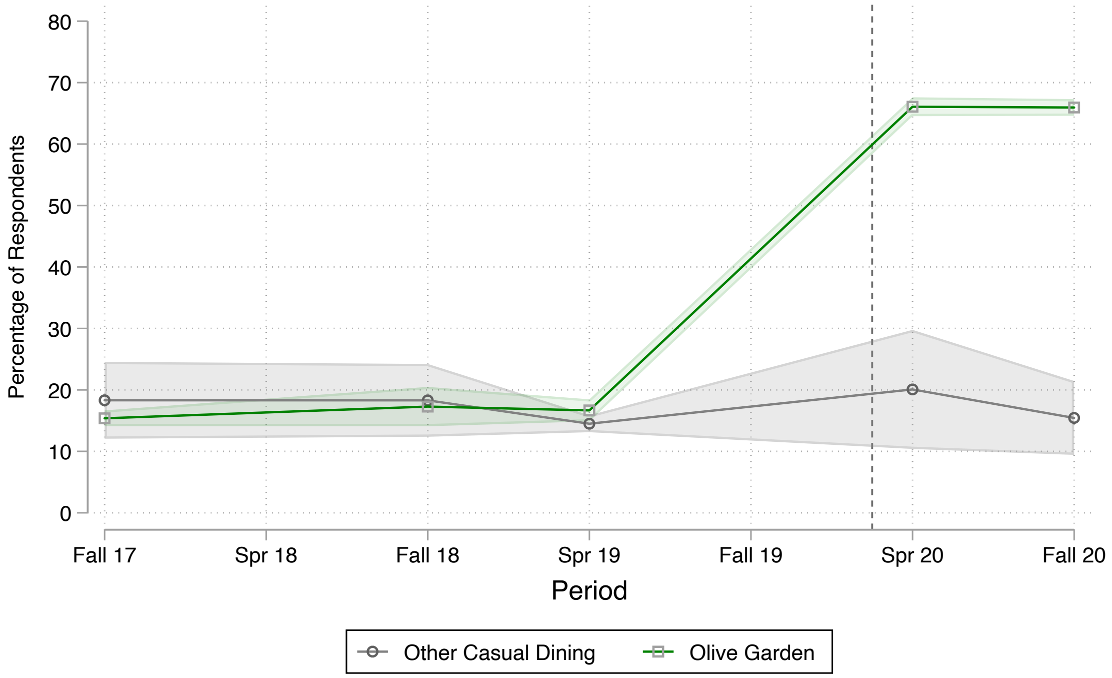

Note: Model is estimated with survey weights and includes controls for gender, race/ethnicity, age, whether the respondent has children, whether the respondent speaks a language other than English at home, whether the respondent is currently enrolled in school, respondent's educational attainment, whether the respondent is married or living with a partner, whether respondent is a manager, how many years a respondent has been at their job, hourly wage, what shift the respondent works, who controls the respondent's schedule, and involuntary part-time status. Vertical dashed gray line designates estimated date of PSL policy change. Estimates are connected, but no data are observed between estimated points.

Supplemental Exhibit 5: Difference-in-Differences Estimates of Paid Sick Leave Access, Robustness

	Have Access to PSL					
	M1	M2	M3	M4	M5	M6
Olive Garden x Fall 2017	(ref)	(ref)	(ref)	(ref)	(ref)	(ref)
	(ref)	(ref)	(ref)	(ref)	(ref)	(ref)
Olive Garden x Fall 2018	0.192	0.004	-0.008	0.022	0.033	-0.0002
	(0.038)	(0.082)	(0.070)	(0.065)	(0.072)	(0.050)
Olive Garden x Spring 2019	0.511	0.058	0.056	0.080	0.047	0.061
	(0.027)	(0.079)	(0.063)	(0.056)	(0.040)	(0.039)
Olive Garden x Spring 2020	0.489****	0.552****	0.518****	0.513****	0.485****	0.518****
	(0.058)	(0.073)	(0.063)	(0.063)	(0.054)	(0.054)
Olive Garden x Fall 2020	0.534****	0.555****	0.544****	0.488****	0.468****	0.405****
	(0.267)	(0.074)	(0.062)	(0.067)	(0.062)	(0.052)
Comparison Group	Casual Dining	All Food	All Food	All Food	All Food	All Food
Demographic Controls	Yes	No	Yes	No	Yes	Yes
Work Controls	Yes	No	No	Yes	Yes	Yes
Weights	Weight_1	Weight_1	Weight_1	Weight_1	Weight_2	Unweighted
N	2,562	10,306	10,306	10,306	10,306	10,306

Note: Demographic controls: gender, race/ethnicity, age, whether the respondent has children, whether the respondent speaks a language other than English at home, whether the respondent is currently enrolled in school, respondent's educational attainment, and whether the respondent is married or living with a partner. Work controls: whether respondent is a manager, how many years a respondent has been at their job, hourly wage, what shift the respondent works, who controls the respondent's schedule, if respondent is involuntarily part-time. Weight_1 adjusts for age, race/ethnicity, education, gender, and sub-sector. Weight_2 additionally adjusts for employer size.
* $p < 0.10$; ** $p < 0.05$; *** $p < 0.01$; **** $p < 0.001$

Supplemental Exhibit 6: Difference-in-Difference Estimates of Presenteeism at Olive Garden versus Other Casual Dining Restaurants

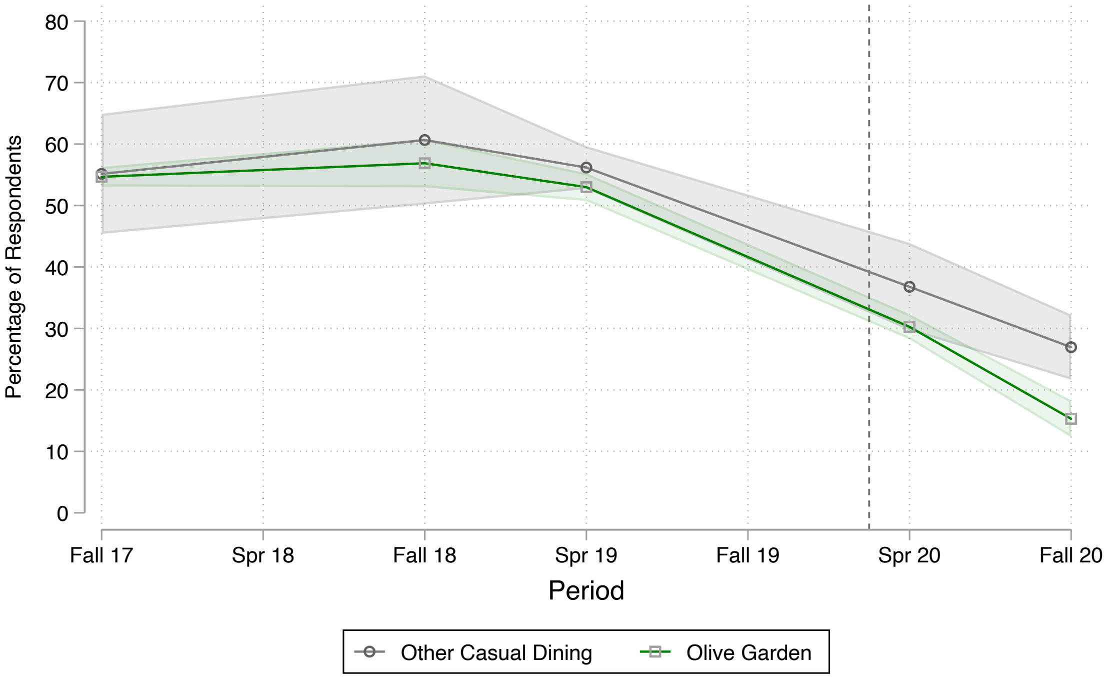

Note: Model is estimated with survey weights and includes controls for gender, race/ethnicity, age, whether the respondent has children, whether the respondent speaks a language other than English at home, whether the respondent is currently enrolled in school, respondent's educational attainment, whether the respondent is married or living with a partner, whether respondent is a manager, how many years a respondent has been at their job, hourly wage, what shift the respondent works, who controls the respondent's schedule, and involuntary part-time status. Vertical dashed gray line designates estimated date of PSL policy change. Estimates are connected, but no data are observed between estimated points

Supplemental Exhibit 7: Difference-in-Differences Estimates of Presenteeism, Robustness

	Presenteeism					
	M1	M2	M3	M4	M5	M6
Olive Garden x Fall 2017	(ref)	(ref)	(ref)	(ref)	(ref)	(ref)
	(ref)	(ref)	(ref)	(ref)	(ref)	(ref)
Olive Garden x Fall 2018	-0.034	-0.018	-0.019	-0.032	-0.038	0.045**
	(0.062)	(0.027)	(0.027)	(0.028)	(0.026)	(0.017)
Olive Garden x Spring 2019	-0.007	-0.027	-0.031	-0.035	-0.046	-0.039
	(0.043)	(0.033)	(0.033)	(0.035)	(0.029)	(0.024)
Olive Garden x Spring 2020	-0.037	-0.056	-0.060	-0.046	-0.027	0.024
	(0.057)	(0.038)	(0.035)	(0.036)	(0.024)	(0.026)
Olive Garden x Fall 2020	-0.096*	-0.173****	-0.171****	-0.149****	-0.136****	-0.074**
	(0.048)	(0.040)	(0.037)	(0.038)	(0.030)	(0.031)
Comparison Group	Casual Dining	All Food	All Food	All Food	All Food	All Food
Demographic Controls	Yes	No	Yes	No	Yes	Yes
Work Controls	Yes	No	No	Yes	Yes	Yes
Weights	Weight_1	Weight_1	Weight_1	Weight_1	Weight_2	Unweighted
N	2,562	10,306	10,306	10,306	10,306	10,306

Note: Demographic controls: gender, race/ethnicity, age, whether the respondent has children, whether the respondent speaks a language other than English at home, whether the respondent is currently enrolled in school, respondent's educational attainment, and whether the respondent is married or living with a partner. Work controls: whether respondent is a manager, how many years a respondent has been at their job, hourly wage, what shift the respondent works, who controls the respondent's schedule, if respondent is involuntarily part-time. Weight_1 adjusts for age, race/ethnicity, education, gender, and sub-sector. Weight_2 additionally adjusts for employer size.
* $p < 0.10$; ** $p < 0.05$; *** $p < 0.01$; **** $p < 0.001$
